# ﻿Species diversity of *Tricholoma* (Agaricales, Tricholomataceae) from Shanxi Province of northern China with the description of four new species

**DOI:** 10.3897/mycokeys.112.132652

**Published:** 2025-01-09

**Authors:** Ning Mao, Jia-Jia Yang, Yu-Xin Zhang, Ting Li, Li Fan

**Affiliations:** 1 College of Life Science, Capital Normal University, Xisanhuanbeilu 105, Haidian, Beijing 100048, China Capital Normal University Beijing China; 2 Department of Life Sciences, Natural History Museum of China, Tianqiaonandajie126, Dongcheng, Beijing, 100050, China Natural History Museum of China Beijing China

**Keywords:** Basidiomycota, ectomycorrhiza, phylogenetic analyses, taxonomy

## Abstract

Species of *Tricholoma* are of great economic and ecological value. There are many studies on *Tricholoma* worldwide, but the areas investigated are generally North America and Europe. There is limited knowledge about *Tricholoma* in China. In this study, 21 species of *Tricholoma* were confirmed in Shanxi Province based on morphological and molecular phylogenetic analyses. These species are located in eight sections, viz. sect. Atrosquamosa, sect. Genuina, sect. Lasciva, sect. Matsutake, sect. Pardinicuti, sect. Rigida, sect. Terrea and sect. Tricholoma. Of these, four species are described as new to science: *T.flavoviride*, *T.fumeobrunneum*, *T.parafulvum*, and *T.viscidum*.

## ﻿Introduction

*Tricholoma* (Fr.) Staude was erected as a genus in the year 1857 (Staude 1857). This genus is mainly characterized by fleshy basidiomata, adnexed to emarginate lamellae, a central stipe, white spore prints, subglobose to oblong basidiospores, simple pileipellis structures and often the absence of well-differentiated cystidia (Kost 1981; Singer 1986; Christensen and Noordeloos 1999; Bessette et al. 2013; Christensen and Heilmann-Clausen 2013; Heilmann-Clausen et al. 2017; Reschke et al. 2018; Ding et al. 2023). Species of *Tricholoma* are ecologically and economically vital, not only for forming ectomycorrhizal symbioses with trees of the families Pinaceae, Betulaceae, Fagaceae, Salicaceae, Myrtaceae and Nothofagaceae, but also as famous delicacy mushrooms, such as *T.matsutake* (S. Ito & S. Imai) Singer and its allies (Bougher 1996; Chapela and Garbelotto 2004; Matsushita et al. 2005; Suzuki 2005; Tedersoo et al. 2010; Ota et al. 2012; Bessette et al. 2013; Christensen and Heilmann-Clausen 2013; Sánchez-García and Matheny 2016; Heilmann-Clausen et al. 2017; Reschke et al. 2018; Ding et al. 2023). Based on morphological and molecular approaches, a number of infrageneric classifications of *Tricholoma* have been proposed (Bon 1984, 1991; Singer 1986; Riva 1988, 2003; Christensen and Noordeloos 1999; Noordeloos and Christensen 1999; Christensen and Heilmann-Clausen 2008; Reschke et al. 2018; Landry et al. 2022; Trudell et al. 2022; Ding et al. 2023). For example, Ding et al. (2023) assessed subgenera and section boundaries within *Tricholoma* by using morphology and phylogenetic evidence from 50-locus, in which a total of four subgenera and 11 sections were identified.

Recently, some important taxonomic works have been focused on *Tricholoma* in China (Cui et al. 2022; Ding et al. 2023; Yang et al. 2023). However, our knowledge of *Tricholoma* is significantly insufficient in Shanxi Province. During the last seven years, we collected a lot of specimens of *Tricholoma* in this region. Our morphological examinations and molecular analyses based on these collections showed they represented 21 species, including four un­described species. The aim of this paper is to describe these new species and clarify the species diversity and geographic distribution of *Tricholoma* in Shanxi Province, northern China.

## ﻿Materials and methods

### ﻿Morphological studies

Collections were obtained and photographed in the field from Shanxi Province, North China, dried in a fruit drier at 40–45 °C, and deposited at BJTC (Herbarium Biology Department, Capital Normal University) and HSA (Herbarium of Shanxi Institute for Functional Foods, Shanxi Agricultural University). Macroscopic characters were recorded from fresh specimens. Standardized color values matching the color of the description were taken from ColorHexa (http://www.colorhexa.com/). Microscopic characteristics were observed in sections obtained from dry specimens mounted in 5% KOH, Congo Red, or Melzer’s reagent (Dring 1971). Dimensions of basidiospores are given using the following format ‘(a–)b–c(–d)’, where the range ‘b–c’ represents at least 90% of the measured values, and ‘a’ and ‘d’ are the most extreme values. ‘Q’ refers to the length/width ratio of basidiospores in the side view.

### ﻿DNA extraction, PCR amplification, and DNA sequencing

A small amount of basidiomata material (20–30 mg) was crushed by shaking for 45 seconds at 30 Hz 2–4 times (Mixer Mill MM301, Haan, Germany) in a 1.5 mL tube, together with a 3 mm diam. tungsten carbide ball. Total genomic DNA was extracted from the powdered basidiomata using NuClean Plant Genomic DNA Kit (CWBIO, Beijing, China), following the manufacturer’s instructions. The ITS region was amplified using the primers ITS1-F/ITS4 (White et al. 1990; Gardes and Bruns 1993). Polymerase Chain Reactions (PCR) for ITS region was performed in 25 µl reaction containing 2 μl DNA template, 1 µl primer (10 µM) each, 12.5 µl of 2 × Master Mix [Tiangen Biotech (Beijing) Co.], 8.5 μl ddH2O. PCR reactions were implemented as follows: an initial denaturation at 94 °C for 5 min; then 35 cycles of the following denaturation at 94 °C for 30 s, annealing at 55 °C for 45 s, 72 °C for 1 min; and a final extension at 72 °C for 10 min. The PCR products were sent to Beijing Zhongkexilin Biotechnology Co. Ltd. (Beijing, China) for purifying, sequencing, and editing. Accession numbers of new and downloaded sequences stored in the NCBI database are provided in Table 1.

**Table 1.** Specimens used in ITS phylogenetic analysis and their GenBank accession numbers. Newly generated sequences are in bold.

Taxon name	Specimen voucher	Origin	ITS
*Leucopaxilluslaterarius*	KUN-HKAS 106319	China	MW724394
*Leucopaxillustricolor*	MB 000946	Germany	MW724429
*Tricholomaalbobrunneum*	MC99-060	France	LT000077
*Tricholomaalbobrunneum*	KUN-HKAS 68189	China	MW724479
*Tricholomaalbum*	MC95-159	Denmark	LT000008
*Tricholomaalbum*	MB 006366	Germany	MW724416
*Tricholomaalbum*	MB 006323	Germany	MW724421
*Tricholomaammophilum*	WTU-F-073083	USA	MW597140
*Tricholomaammophilum*	WTU-F-073015	USA	MW597199
***Tricholomaammophilum***	**HSA 371**	**China**	**PQ499533**
*Tricholomaargyraceum*	MEN9491	Netherlands	LT000198
***Tricholomaargyraceum***	**BJTC FM2197**	**China**	**PQ499519**
***Tricholomaargyraceum***	**BJTC FM2372**	**China**	**PQ499520**
***Tricholomaatrosquamosum***	**BJTC FM4346**	**China**	**PQ499525**
*Tricholomaaurantium*	MC97-227	Denmark	LT000012
***Tricholomaaurantium***	**BJTC FM2193**	**China**	**PQ499534**
***Tricholomaaurantium***	**BJTC FM2195**	**China**	**PQ499535**
“*Tricholomaauratum*”	Tk1	Japan	AB289663
“*Tricholomaauratum*”	ISK1	Japan	AB289662
*Tricholomabadicephalum*	UBC-F-16235r	Canada	MW597207
*Tricholomabadicephalum*	WTU-F-073095	USA	MW597309
*Tricholomabakamatsutake*	TNS:F-12866	Japan	AB699654
*Tricholomabakamatsutake*	KUN-HKAS 106313	China	MW724402
*Tricholomabakamatsutake*	KUN-HKAS 107570	China	MW724468
***Tricholomabakamatsutake***	**BJTC FM3440**	**China**	**PQ499506**
***Tricholomabakamatsutake***	**BJTC FM3441**	**China**	**PQ499507**
*Tricholomabasirubens*	MC01-209	Croatia	LT000001
*Tricholomabasirubens*	TL5303	Sweden	LT000158
*Tricholomabatschii*	KMS436	USA	AF377238
*Tricholomabatschii*	MB-003027	Germany	MF034298
*Tricholomabonii*	LUG-F8450	Italy	LT000101
*Tricholomabonii*	HMJAU35946	China	MW724393
***Tricholomabonii***	**BJTC FM1280**	**China**	**PQ499508**
***Tricholomabonii***	**BJTC FM1388**	**China**	**PQ499509**
***Tricholomabonii***	**BJTC FM920**	**China**	**PQ499510**
*Tricholomaboudieri*	HKAS97070	China	MW724437
*Tricholomaboudieri*	HKAS97163	China	MW724373
***Tricholomaboudieri***	**BJTC FM1528**	**China**	**PQ499521**
***Tricholomaboudieri***	**BJTC FM2752**	**China**	**PQ499522**
*Tricholomacingulatum*	MC96-134	Denmark	LT000015
***Tricholomacingulatum***	**BJTC FM1172**	**China**	**PQ499516**
***Tricholomacingulatum***	**BJTC FM820**	**China**	**PQ499517**
***Tricholomacingulatum***	**BJTC FM1191**	**China**	**PQ499518**
*Tricholomacitrinum*	MB-305716	China	MF034262
*Tricholomacitrinum*	KUN-HKAS 71086	China	MW724356
*Tricholomacolossus*	MC97-047	Sweden	LT000164
*Tricholomacolossus*	MB-002363	Germany	MF034285
*Tricholomacolumbetta*	MC95-181	Denmark	LT000017
*Tricholomacolumbetta*	MQ20-HRL3139-QFB32663	Canada	MW628118
*Tricholomadulciolens*	H:7002022	Sweden	AB738883
*Tricholomadulciolens*	-	USA	AF309523
*Tricholomaelegans*	OTA:61947	New Zealand	JX178630
*Tricholomaelegans*	TENN:063711	New Zealand	KJ417316
*Tricholomaequestre*	MC94-027	Denmark	LT000018
*Tricholomaequestre*	MC96-155	Denmark	LT000020
*Tricholomaequestre*	HMJAU22249	Belarus	MW724392
“*Tricholomaequestre*”	MB305549	China	MF034257
“*Tricholomaequestre*”	MB305676	China	MF034261
“*Tricholomaequestre*”	MB-301506	China	MF034239
“*Tricholomaequestre*”	EqFrW	France	HM590874
*Tricholomafilamentosum*	C-F-35924	Sweden	LT000165
*Tricholomafilamentosum*	MB 000950 (KR9404)	Germany	MW724422
“*Tricholomaflavovirens*”	trh545	USA	AF458449
“*Tricholomaflavovirens*”	trh546	USA	AF458452
“*Tricholomaflavovirens*”	613	Japan	AB036895
“*Tricholomaflavovirens*”	KGP52	USA	DQ822834
***Tricholomaflavoviride***	**BJTC FM3966**	**China**	**PQ499559**
***Tricholomaflavoviride***	**BJTC FM4164**	**China**	**PQ499560**
***Tricholomaflavoviride***	**BJTC FM3512**	**China**	**PQ499561**
*Tricholomafocale*	JV97-239	Sweden	LT000166
*Tricholomafocale*	KUN-HKAS 106309	China	MW724460
*Tricholomaforteflavescens*	HKAS93511	China	MF034207
*Tricholomaforteflavescens*	MB-301985	China	MF034246
*Tricholomafrondosae* type I	MC95-130	Sweden	LT000167
*Tricholomafrondosae* type I	KUN-HKAS 98072	China	MW724365
*Tricholomafrondosae* type I	KUN-HKAS 87149	China	MW724346
*Tricholomafrondosae* type II	MC96-235	Denmark	LT000023
*Tricholomafrondosae* type II	MC00-225	Slovenia	LT000140
***Tricholomafrondosae* type II**	**BJTC FM2026**	**China**	**PQ499556**
***Tricholomafrondosae* type II**	**BJTC FM3895**	**China**	**PQ499557**
***Tricholomafrondosae* type II**	**BJTC FM4157**	**China**	**PQ499558**
*Tricholomafulvomaculatum*	HKAS107572	China	MW724472
*Tricholomafulvomaculatum*	HKAS107576	China	MW724473
*Tricholomafulvum*	JHC04-251	Sweden	LT000171
*Tricholomafulvum*	MQ20-YL-CMMF001495	Canada	MW627880
***Tricholomafumeobrunneum***	**BJTC FM3445**	**China**	**PQ499552**
***Tricholomafumeobrunneum***	**BJTC FM3436**	**China**	**PQ499553**
***Tricholomafumeobrunneum***	**BJTC FM3571**	**China**	**PQ499554**
***Tricholomafumeobrunneum***	**BJTC FM3574**	**China**	**PQ499555**
*Tricholomahighlandense*	HKAS70192	China	KY488549
*Tricholomahighlandense*	KUN-HKAS 107590	China	MW724452
*Tricholomailkkae*	S-F173364	Sweden	LT222028
*Tricholomailkkae*	S-F513823	Sweden	LT222029
“*Tricholomajoachimii*”	O-F167194	Norway	LT222022
*Tricholomaimbricatum*	KUN-HKAS 112559	China	MW724476
*Tricholomaimbricatum*	KUN-HKAS 87886	China	MW724327
***Tricholomaimbricatum***	**BJTC FM1170**	**China**	**PQ499549**
***Tricholomaimbricatum***	**BJTC FM1445**	**China**	**PQ499550**
***Tricholomaimbricatum***	**BJTC FM1446**	**China**	**PQ499551**
*Tricholomainocybeoides*	MC03-229	Denmark	LT000025
*Tricholomainocybeoides*	MC97-060	Sweden	LT000176
*Tricholomalascivum*	MC00-519	Denmark	LT000028
*Tricholomalascivum*	MB-303096	Ukraine	MF034316
***Tricholomalishanense***	**BJTC FM1023**	**China**	**PQ499526**
***Tricholomalishanense***	**BJTC FM1137**	**China**	**PQ499527**
***Tricholomalishanense***	**BJTC FM1735**	**China**	**PQ499528**
*Tricholomamatsutake*	KUN-HKAS 98323	China	MW724385
*Tricholomamatsutake*	MC03-600	Sweden	LT000178
*Tricholomamatsutake*	TNS:F-12850	Japan	AB699630
*Tricholomamurrillianum*	SAT-16-319-01	USA	KY660032
*Tricholomamurrillianum*	NY586560	USA	LT220179
*Tricholomaolivaceotinctum*	MC97103	Sweden	FJ544861
*Tricholomaolivaceotinctum*	KUN-HKAS 107586	China	MW724405
*Tricholomaolivaceum*	HKAS93513	China	MF034209
*Tricholomaolivaceum*	KUN-HKAS 68600	China	MW724351
*Tricholomaorienticolossus*	HAKS99341	China	MT124443
*Tricholomaorienticolossus*	HAKS98045	China	MT124444
*Tricholomaorientifulvum*	HAKS107157	China	MT114682
*Tricholomaorientifulvum*	HAKS107156	China	MT124445
***Tricholomaparafulvum***	**BJTC FM2500**	**China**	**PQ499536**
***Tricholomaparafulvum***	**BJTC FM4065**	**China**	**PQ499537**
***Tricholomaparafulvum***	**BJTC FM4210**	**China**	**PQ499538**
*Tricholomapardinum*	C-F-96190	USA	LT000142
*Tricholomapardinum*	MB 006381	Germany	MW724424
*Tricholomapessundatum*	JV04-482	Denmark	LT000032
*Tricholomapessundatum*	MQ20-JLAB931-CMMF009347	Canada	MW628012
*Tricholomapopulinum*	O-F63960	Norway	JN019594
*Tricholomapopulinum*	KUN-HKAS 106657	China	MW724411
***Tricholomapopulinum***	**BJTC FM1144**	**China**	**PQ499529**
***Tricholomapopulinum***	**BJTC FM1145**	**China**	**PQ499530**
***Tricholomapopulinum***	**BJTC FM1165**	**China**	**PQ499531**
***Tricholomapopulinum***	**BJTC FM1979**	**China**	**PQ499532**
*Tricholomapsammopus*	MC04-600	Slovenia	LT000145
*Tricholomapsammopus*	KUN-HKAS 106302	China	MW724436
***Tricholomapsammopus***	**BJTC FM2673**	**China**	**PQ499546**
***Tricholomapsammopus***	**BJTC FM1405**	**China**	**PQ499547**
***Tricholomapsammopus***	**BJTC FM814**	**China**	**PQ499548**
*Tricholomaqiaomianjun*	KUN-HKAS 101303	China	OK036719
*Tricholomaqiaomianjun*	KUN-HKAS 115901	China	OK036720
*Tricholomaroseoacerbum*	MQ20-HRL1010a-QFB32619	Canada	MW628060
*Tricholomaroseoacerbum*	KUN-HKAS 88046	China	MW724332
*Tricholomarufobrunneum*	KUN-HKAS49069	China	OL331894
*Tricholomarufobrunneum*	KUN-HKAS90808	China	OL331895
*Tricholomasaponaceum*	C-F23337	Denmark	LT000038
*Tricholomasaponaceum*	JHC00-049	Norway	LT000123
*Tricholomasaponaceum*	MB-002941	Germany	MF034221
***Tricholomasaponaceum***	**BJTC FM2772**	**China**	**PQ499523**
***Tricholomasaponaceum***	**BJTC FM2773**	**China**	**PQ499524**
*Tricholomasinoacerbum*	GDGM:44680	China	KT160219
*Tricholomasinoacerbum*	KUN-HKAS 105349	China	MW724434
*Tricholomasinopardinum*	KUN-HKAS 91129	China	MW724361
*Tricholomasinopardinum*	HKAS82533	China	KY488552
*Tricholomasmithii*	DBG:CLO4513	USA	MG719957
*Tricholoma* sp.	HKAS106303	China	MW724450
*Tricholoma* sp.	HKAS101281	China	MW724443
*Tricholomasquarrulosum*	JHC93-224	Denmark	LT000047
*Tricholomasquarrulosum*	JHC93-262	Denmark	LT000048
*Tricholomastans*	MC95-145	Sweden	LT000189
*Tricholomastans*	KUN-HKAS 87940	China	MW724329
*Tricholomastiparophyllum*	MC95-117	Sweden	LT000190
*Tricholomastiparophyllum*	MQ20-GUE1522-CMMF014811	Canada	MW628089
*Tricholomasudum*	JV96-306	Denmark	LT000050
*Tricholomasudum*	MC98-601	Denmark	LT000051
*Tricholomaterreum*	MEN95192	Germany	LT000098
*Tricholomaterreum*	KUN-HKAS 69914	China	MW724459
***Tricholomaterreum***	**BJTC FM2414**	**China**	**PQ499511**
***Tricholomaterreum***	**BJTC FM3657**	**China**	**PQ499512**
***Tricholomaterreum***	**BJTC FM3677**	**China**	**PQ499513**
*Tricholomatriste*	E3754	Germany	LT000099
***Tricholomatriste***	**BJTC FM1269**	**China**	**PQ499514**
***Tricholomatriste***	**BJTC FM3817**	**China**	**PQ499515**
“*Tricholomaulvinenii*”	JuV13229F	Finland	LT000068
“*Tricholomaulvinenii*”	JuV26740F	Finland	LT000069
“*Tricholomaulvinenii*”	IK931613	Finland	LT000067
*Tricholomaumbonatum* type I	MC00A01	Denmark	LT000063
*Tricholomaumbonatum* type II	TRgmb00651	Italy	LT000114
*Tricholomaustale*	JHC92-299	Denmark	LT000064
*Tricholomaustale*	MB-002924	Germany	MF034288
*Tricholomaustaloides*	MC99-067	France	LT000094
*Tricholomaustaloides*	MB-002929	Germany	MF034291
*Tricholomavaccinum*	MC95-109	Sweden	LT000195
*Tricholomavaccinum*	DBG:23466	USA	MF034272
*Tricholomavaccinum*	KUN-HKAS 98065	China	MW724364
***Tricholomavaccinum***	**BJTC FM937**	**China**	**PQ499543**
***Tricholomavaccinum***	**BJTC FM943**	**China**	**PQ499544**
***Tricholomavaccinum***	**BJTC FM1425**	**China**	**PQ499545**
*Tricholomavenenatoides*	WTU-F-073089	USA	MW597303
***Tricholomaviscidum***	**BJTC FM4198**	**China**	**PQ499539**
***Tricholomaviscidum***	**BJTC FM3388**	**China**	**PQ499540**
***Tricholomaviscidum***	**BJTC FM4038**	**China**	**PQ499541**
***Tricholomaviscidum***	**BJTC FM4199**	**China**	**PQ499542**

### ﻿Phylogenetic analyses

For this study, the ITS dataset was compiled to identify the new species and to investigate their phylogenetic position in the *Tricholoma*. *Leucopaxillustricolor* (Peck) Kühner and *Leucopaxilluslaterarius* (Peck) Singer & A.H. Sm. were selected as outgroups based on previous studies (Ding et al. 2023). The sequences of the ITS were aligned in the online version of MAFFT 7.110 using default parameters (Katoh and Standley 2013) and manually edited in BioEdit v.7.0.9 (Hall 1999). The final alignments were submitted to TreeBASE 31776.

Phylogenetic analyses were conducted using maximum likelihood (ML) and Bayesian inference (BI). ML analysis was carried out in RAxML 8.0.14 (Stamatakis 2014) with parameters at default settings using a GTRGAMMAI model. 1000 bootstrap replicates were computed in RAxML using a rapid bootstrap analysis and search for the best-scoring ML tree. BI analysis was performed in MrBayes 3.1.2 (Ronquist and Huelsenbeck 2003). The best-fitted substitution model for ITS was determined through MrModeltest v2.3 (Nylander 2004) by using the Akaike Information Criterion (AIC). GTR+I+G was chosen as the best model for ITS. We used two independent runs with four Markov chains Monte Carlo (MCMC) for 3 775 000 generations under the default settings. The average standard deviations of split frequency (ASDSF) values were far lower than 0.01 at the end of the runs. Trees were sampled every 100 generations after burn-in (25% of trees were discarded as the burn-in phase of the analyses, set up well after convergence), and 50% of majority-rule consensus trees were constructed.

Clades with bootstrap support (MLBS) ≥ 70% and Bayesian posterior probability (BPP) ≥ 0.95 were considered significantly supported (Hillis and Bull 1993; Alfaro et al. 2003). All phylogenetic trees were viewed with TreeView (Page 2001).

## ﻿Results

### ﻿Phylogenetic analyses

**Figure 1.**
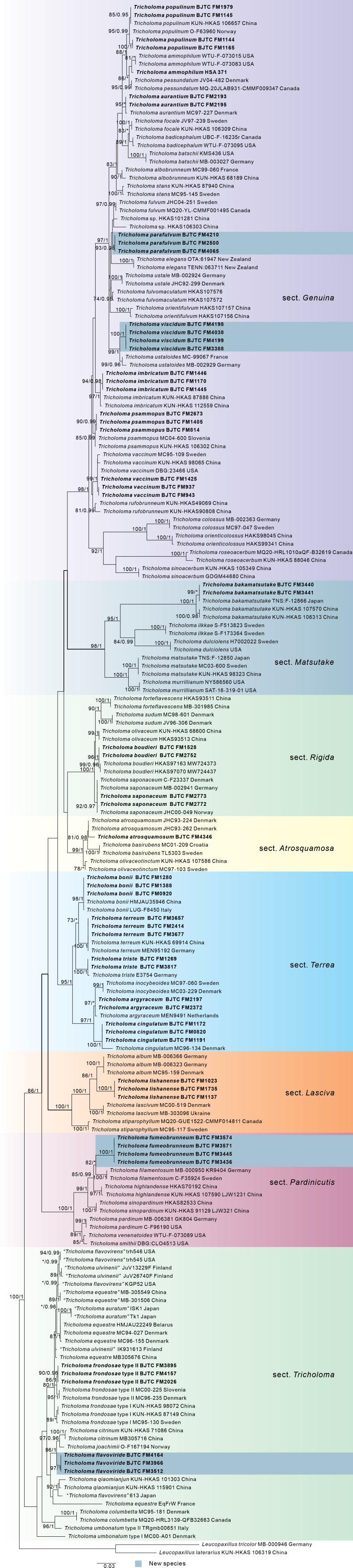
Phylogenetic tree generated from a maximum likelihood analysis based on ITS sequences, showing the phylogenetic relationships of *Tricholoma*. Numbers representing likelihood bootstrap support (MLB ≥ 70%, left) and significant Bayesian posterior probability (BPP ≥ 0.95, right) are indicated above the nodes. Novel sequences are printed in bold.

The ITS dataset consisted of 191 sequences, including 56 sequences newly generated from our collections. The length of the dataset was 578 bp after the exclusion of poorly aligned sites. Our present analyses (Fig. 1) revealed that members of *Tricholoma* in this dataset formed a monophyletic lineage with high likelihood bootstrap support (MLB = 100%) and strong posterior probability support (BPP = 1.00). The sequences of our 56 collections formed 21 strongly support clades, indicating they were 21 distinct species, and these phylogenetic species were respectively placed in eight sections, i.e. sect. Atrosquamosa, sect. Genuina, sect. Lasciva, sect. Matsutake, sect. Pardinicuti, sect. Rigida, sect. Terrea and sect. Tricholoma. Of them, four species are described as new species (see Taxonomy in this paper), and the remaining 17 clades correspond well to the previously described species (also see the photos of their basidiocarps in Figs 4, 5).

### ﻿Taxonomy

#### ﻿ Tricholoma flavoviride

Taxon classificationFungiAgaricalesTricholomataceae

L. Fan, N. Mao & J.J Yang sp. nov.

02CCC616-12D7-50DA-8E60-F40B1D003F1D

MB 856305

Figs 2A, B , 3A, C

##### Diagnosis.

It is distinguished by the combination of the following features: greenish-yellow to yellowish-brown pileus, stipe surface with brownish fibrils, or reflexed squamules. It is most similar to *T.citrinum* Y.Y. Cui & Zhu L. Yang but differs by habitat associated with broadleaf forest.

##### Holotype.

China • Shanxi Province, Pu County, Wulu Mountain, 36°33'34"N, 111°12'40"E, elev. 1510 m, on the ground in broadleaf forest dominated by *Quercus* sp., 28 September 2023, J.Z Cao, MS808 (BJTC FM4164), GenBank Acc. No.: ITS = PQ499560, mtSSU = PQ499564, *rpb2* = PQ509907, *mcm7* = PQ509917.

##### Etymology.

*flavoviride* (Lat.): referring to the color of the basidiomata.

##### Description.

***Basidiomata*** small to medium-sized. ***Pileus*** 20–55 mm diam., at first hemispherical to convex, later plano-convex to applanate with age, with an umbo at center; surface dry, slightly viscid when wet, greenish-yellow (#dbd252) to yellowish-brown (#c2a677), center darker to dark brown (#7a614d) when mature, covered with brown (#b49976) squamules; margin incurved or not. ***Lamellae*** sinuate, crowded, greenish-yellow (#b4ae6c) to yellowish-brown (#d9c45b); lamellulae in 2–4 tiers, concolorous with lamellae. ***Stipe*** 32–70 mm long, 12–17 mm diam., cylindrical, equal or enlarge downwards, dry, white (#f9f9f8) at apex, pale yellow (#e7e1a5) to yellowish brown (#d7cb67) below, covered with brownish (#9c8c58) fibrils or reflexed squamules. ***Context*** white (#ffffff). ***Odor*** unrecorded. ***Taste*** not recorded.

**Figure 2.**
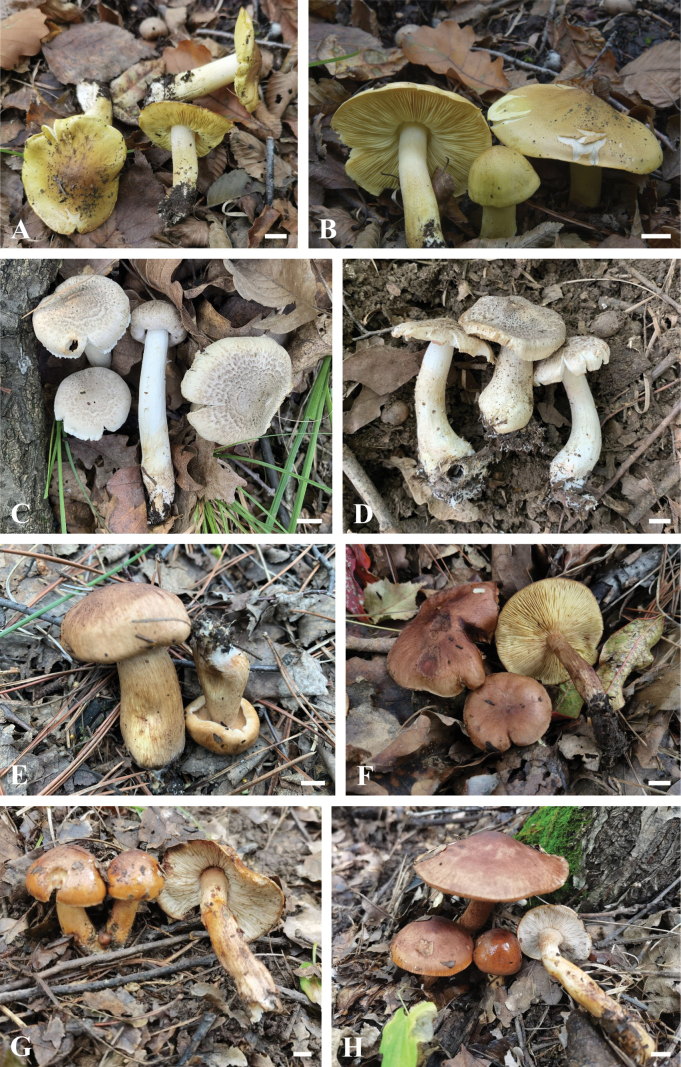
Photographs of basidiomata in their natural habitat **A, B***Tricholomaflavoviride* (**A** BJTC FM3996, **B** BJTC FM4164) **C, D***T.fumeobrunneum* (**C** BJTC FM3571, **D** BJTC FM3574) **E, F***T.parafulvum* (**E** BJTC FM2500, **F** BJTC FM4065) **G, H***T.viscidum* (**G** BJTC FM4038, **H** BJTC FM4198). Scale bars: 1 cm.

***Basidiospores*** [90/3/3] 5–6.5 × 3.5–5 μm, Q = 1.2–1.6, ellipsoid, smooth, inamyloid, usually containing one large oil droplet. ***Basidia*** 24–37 × 5.5–7.5 μm, clavate, (2–)4-spored, sterigmata up to 4.5 μm long. ***Cystidioid cells*** in hymenium absent. ***Hymenophoral trama*** regular, composed of cylindrical hyphae, 4–10 μm wide, colorless in water and 5% KOH. ***Pileipellis*** a cutis, composed of cylindrical hyphae, 4–10 µm wide, colorless or yellowish in water and 5% KOH. ***Stipitipellis*** a cutis, composed of parallel hyphae, 2–6 µm wide, colorless in water and 5% KOH. ***Clamps*** absent in all tissues.

**Figure 3.**
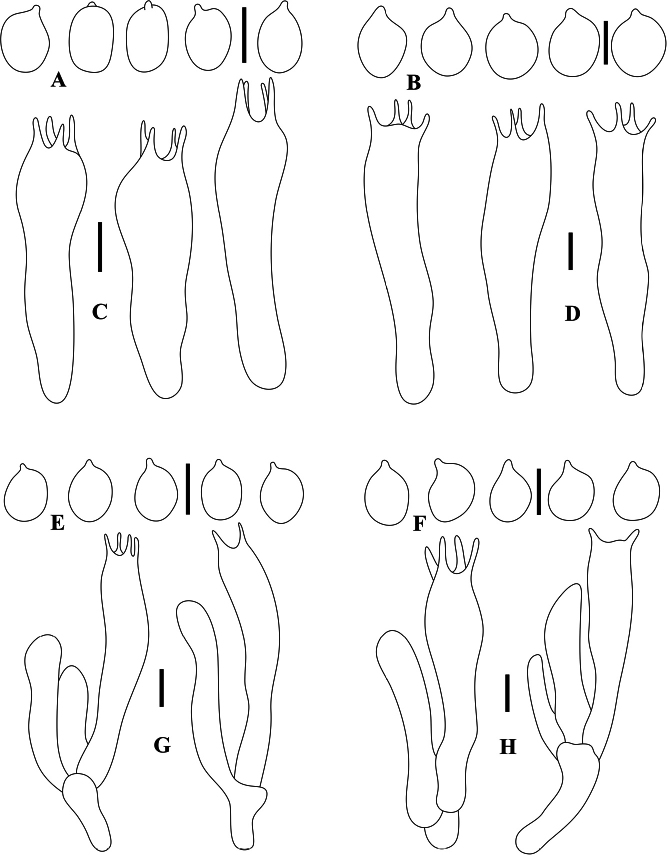
Microscopic features **A, C***Tricholomaflavoviride***B, D***T.fumeobrunneum***E, G***T.parafulvum***F, H***T.viscidum***A, B, E, F** basidiospores **C, D, G, H** basidia. Scale bars: 5 μm.

##### Ecology and habitat.

Scattered or gregarious on the ground in broadleaf forest dominated by *Quercus* sp., currently only known from Shanxi Province, northern China.

**Figure 4.**
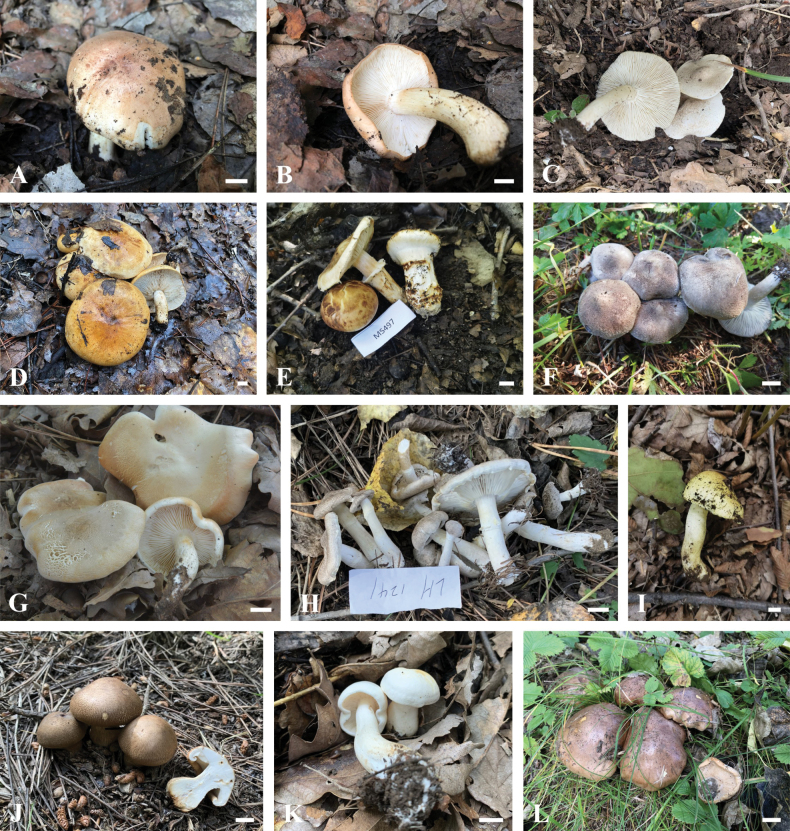
Photographs of basidiomata in their natural habita **A, B***Tricholomaammophilum* (HSA 371) **C***T.argyraceum* (BJTC FM3571) **D***T.aurantium* (BJTC FM2195) **E***T.bakamatsutake* (BJTC FM3441) **F***T.bonii* (BJTC FM905) **G***T.boudieri* (BJTC FM2752) **H***T.cingulatum* (BJTC FM1172) **I***T.frondosae* (BJTC FM3895) **J***T.imbricatum* (BJTC FM1170) **K***T.lishanense* (BJTC FM1735) **L***T.populinum* (BJTC FM1144). Scale bars: 1 cm.

##### Additional specimens examined.

China • Shanxi Province, Pu County, Wulu Mountain, on the ground in broadleaf forest dominated by *Quercus* sp., 28 September 2023, J.Z Cao, CF2196 (BJTC FM3966), GenBank Acc. No.: ITS = PQ499559, mtSSU = PQ499563, *rpb2* = PQ509906, *mcm7* = PQ509916; ibid., Qinshui County, Lishan Mountain, on the ground in broadleaf forest dominated by *Quercus* sp., 7 September 2023, N. Mao, MNM829 (BJTC FM3512), GenBank Acc. No.: ITS = PQ499561, mtSSU = PQ499562, *rpb2* = PQ509905, *mcm7* = PQ509915.

**Figure 5.**
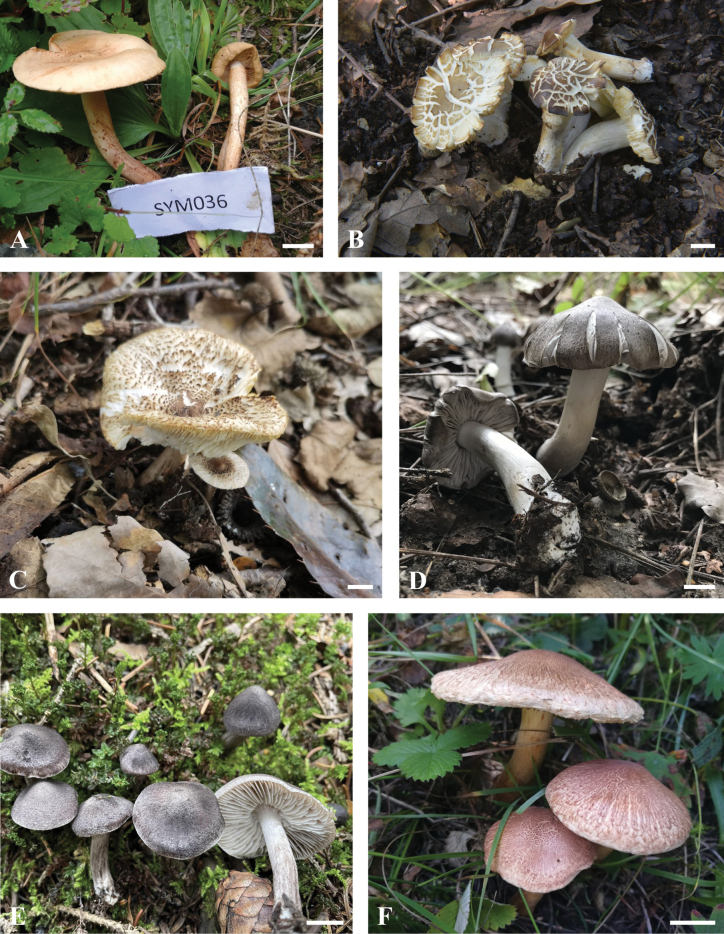
Photographs of basidiomata in their natural habitat **A***Tricholomapsammopus* (BJTC FM814) **B***T.saponaceum* (BJTC FM2773) **C***T.atrosquamosum* (BJTC FM4346) **D***T.terreum* (HSA 419) **E***T.triste* (BJTC FM3817) **F***T.vaccinum* (BJTC FM943). Scale bars: 1 cm.

##### Notes.

*Tricholomaflavoviride* belongs to the sect. Tricholoma (Fig. 1). *Tricholomacitrinum*, a species recently described from Yunnan Province of southwestern China (Cui et al. 2022), is morphologically and phylogenetically closely related to the new species. Both species have brownish-yellow pileus and ellipsoid basidiospores. However, *T.citrinum* is distinguished from *T.flavoviride* by its relatively paler pileus, stipe sometimes smooth, and habitat association with pine forests. Morphologically, *T.flavoviride* is also easily confused with *T.equestre* (L.) P. Kumm., and *T.frondosae* Kalamees & Shchukin. *Tricholomaequestre* is distinguished from *T.flavoviride* by its larger basidiospores (6.5–9 × 4–5.5 μm) and habitat associated with pine forests; *T.frondosae* by its paler pileus and larger basidiospores (7–8.5 × 4–5 μm) (Christensen and Heilmann-Clausen 2013; Reschke et al. 2018; Cui et al. 2022).

#### ﻿ Tricholoma fumeobrunneum

Taxon classificationFungiAgaricalesTricholomataceae

L. Fan, N. Mao & J.J Yang sp. nov.

10FA5221-B489-540B-A811-35C4F5C69C8B

MB 856306

Figs 2C, D , 3B, D

##### Diagnosis.

It is distinguished by the combination of the following features: pale gray to grayish-brown pileus, smooth stipe, and broadly ellipsoid to ellipsoid basidiospores. It is most similar to *T.sinopardinum* Zhu L. Yang, X.X. Ding, G. Kost & Rexer but differs by the smaller basidiospores (6.5–8.5 × 5–6.5 μm).

##### Holotype.

China • Shanxi Province, Lingchuan County, Lishan Mountain, 35°36'6"N, 113°20'32"E, elev. 1185 m, on the ground in broadleaf forest dominated by *Quercus* sp., 28 August 2023, Y. Li, MS492 (BJTC FM3436), GenBank Acc. No.: ITS = PQ499553, mtSSU = PQ499565, *tef1-α* = PQ509897, *rpb2* = PQ509908, *mcm7* = PQ509918.

##### Etymology.

*fumeobrunneum* (Lat.): referring to the cap color of the basidiomata.

##### Description.

***Basidiomata*** small to medium-sized. ***Pileus*** 25–77 mm diam., at first convex, later broadly convex, plano-convex to applanate with age, with an umbo at center; surface dry, pale gray (#cac7c0) to grayish-brown (#9d8166), center darker to dark gray (#aaa9a3) to grayish black (#6b6157) when mature, covered with dark gray (#8e8c95) reflexed fibrillose squamules; margin at first involute, later incurved, cracking with age. ***Lamellae*** sinuate, moderately crowded, dirty white (#f9f8e9) to pale yellow (#f1eeca), turning yellowish brown (#e7dbb2) to brown (#d6bb9c) with age; lamellulae in 2–4 tiers, concolorous with lamellae. ***Stipe*** 50–76 mm long, 7–15 mm diam., cylindrical to clavate, enlarged downwards, dry, white (#f6f7f7), pale yellow (#f1ffe3) to yellowish brown (#ab9065), smooth. ***Context*** white (#f9f9f9), up to 3.3 mm. ***Odor*** unrecorded. ***Taste*** not recorded.

***Basidiospores*** [120/3/4] 6.5–8.5 × 5–6.5 μm, Q = 1.3–1.6, broadly ellipsoid to ellipsoid, smooth, inamyloid, usually containing one large oil droplet. ***Basidia*** 34–46 × 6–9 μm, clavate, (2–)4-spored, sterigmata up to 6 μm long. ***Cystidioid cells*** in hymenium absent. ***Hymenophoral trama*** regular, composed of cylindrical hyphae, 3–12 μm wide, colorless in water and 5% KOH. ***Pileipellis*** a cutis, composed of cylindrical hyphae, 3–10 µm wide, colorless or yellowish-brown in water and 5% KOH. ***Stipitipellis*** a cutis, composed of parallel hyphae, 1.5–6 µm wide, colorless in water and 5% KOH. ***Clamps*** common in all tissues.

##### Ecology and habitat.

Scattered or gregarious on the ground in broadleaf forest dominated by *Quercus* sp., currently only known from Shanxi Province, northern China.

##### Other specimens examined.

China • Shanxi Province, Lingchuan County, Lishan Mountain, on the ground in broadleaf forest dominated by *Quercus* sp., 5 September 2023, N. Mao, MNM891 (BJTC FM3571), GenBank Acc. No.: ITS = PQ499554; ibid., Lingchuan County, Lishan Mountain, 35°36'6"N, 113°20'32"E, elev. 1190 m, on the ground in a broadleaf forest dominated by *Quercus* sp., 28 August 2023 H.M. Ji, MS492 (BJTC FM3445), GenBank Acc. No.: ITS = PQ499552, mtSSU = PQ499566, *tef1-α* = PQ509898, *rpb2* = PQ509909, *mcm7* = PQ509919; ibid., 5 September 2023, N. Mao, MNM894 (BJTC FM3574), GenBank Acc. No.: ITS = PQ499555, mtSSU = PQ499567, *tef1-α* = PQ509899, *mcm7* = PQ509920.

##### Notes.

*Tricholomafumeobrunneum* belongs to the sect. Pardinicutis (Fig. 1). *Tricholomafumeobrunneum* was phylogenetically sister to *T.sinopardinum*, a species found in Xizang Autonomous, southwestern China. However, *T.sinopardinum* differs from the new species by its stipe covered with brownish, brown to dark brown fibrillose to reflexed squamules and larger basidiospores (8.5–10.5 × 6.5–7.5 μm) (Yang et al. 2017). *Tricholomahighlandense* Zhu L. Yang, X.X. Ding, G. Kost & Rexer, another species from China in this section, is easily distinguished from *T.fumeobrunneum* by its pileus without a tinge of gray and occurrence in red acid soil (Yang et al. 2017).

#### ﻿ Tricholoma parafulvum

Taxon classificationFungiAgaricalesTricholomataceae

L. Fan, N. Mao & J.J Yang sp. nov.

34FAB728-E8AE-52C7-A6C0-22565001A50B

MB 856307

Figs 2E, F , 3E, G

##### Diagnosis.

It is distinguished by the combination of the following features: yellowish brown, brown to dark brown pileus, stipe surface covered with brown fibrils, and broadly ellipsoid to ellipsoid basidiospores. It is most similar to *Tricholomafulvum* (DC.) Bigeard & H. Guill. but differs in the pileus surface without being radially streaky.

##### Holotype.

China • Shanxi Province, Qinshui County, Lishan Mountain, 35°36'5"N, 113°20'29"E, elev. 1600 m, on the ground in broadleaf forest dominated by *Quercus* sp., 5 October 2023, J.Z. Cao, CF2300 (BJTC FM4065), GenBank Acc. No.: ITS = PQ499537, mtSSU = PQ499568, *tef1-α* = PQ509900, *rpb2* = PQ509910, *mcm7* = PQ509921.

##### Etymology.

*Parafulvum* (Lat.): referring to the fact that the new species is very similar to *Tricholoma fulvum* in basidiomatal appearance.

##### Description.

***Basidiomata*** small to medium-sized. ***Pileus*** 30–66 mm diam., at first hemispherical to convex, later plano-convex to applanate with age, with depression or an umbo at center; surface dry, slightly viscid when wet, yellowish brown (#edd6b8), brown (#d49d76) to dark brown (#b78877), darker in the center and paler towards the margin, covered with brown (#a1735b) to dark brown (#845546) reflexed fibrillose squamules; margin at first involute, later incurved, cracking with age. ***Lamellae*** sinuate, moderately crowded, yellow (#f3edd7) to yellowish brown (#ddb68b) when young, turning dark brown (#c1986e) with age; lamellulae in 2–3 tiers, concolorous with lamellae. ***Stipe*** 42–83 mm long, 5–22 mm diam., cylindrical to clavate, enlarge downwards, dry, white (#f5fae3), yellowish brown (#c1a36f) to brown (#9d7963), covered with brown (#7e5c43) fibrils. ***Context*** white (#f7f8f7), up to 11 mm. ***Odor*** unrecorded. ***Taste*** not recorded.

***Basidiospores*** [90/3/3] 4.5–6 × 3.5–4.5 μm, Q = 1.3–1.4, broadly ellipsoid to ellipsoid, smooth, inamyloid, usually containing one large oil droplet. ***Basidia*** 23–37 × 4.5–7 μm, clavate, (2–)4-spored, sterigmata up to 5.5 μm long. ***Cystidioid cells*** in hymenium absent. Hymenophoral trama regular, composed of cylindrical hyphae, 3–7 μm wide, colorless in water and 5% KOH. ***Pileipellis*** a cutis, composed of cylindrical hyphae, 3–8 µm wide, colorless or yellowish-brown in water and 5% KOH. ***Stipitipellis*** a cutis, composed of parallel hyphae, 2–7 µm wide, colorless in water and 5% KOH. ***Clamps*** absent in all parts of basidioma.

##### Ecology and habitat.

Scattered or gregarious on the ground in broadleaf forest dominated by *Quercus* sp. or *Betula* sp., currently only known from Shanxi Province, northern China.

##### Additional specimens examined.

China • Shanxi Province, Qinshui County, Lishan Mountain, on the ground in broadleaf forest dominated by *Quercus* sp., 5 October 2023, J.Z. Cao, MS855 (BJTC FM4210), GenBank Acc. No.: ITS = PQ499538, mtSSU = PQ499569, *tef1-α* = PQ509901, *rpb2* = PQ509911, *mcm7* = PQ509922; ibid., Loufan County, Yundingshan Mountain, on the ground in broadleaf forest dominated by *Betula* sp., 23 August 2022, N. Mao, MNM738 (BJTC FM2500), GenBank Acc. No.: ITS = PQ499536.

##### Notes.

*Tricholomaparafulvum* belongs to sect. Genuina (Fig. 1). *Tricholomafulvum* is morphologically similar and phylogenetically closely related to the new species. They all have brownish caps and ellipsoid basidiospores. However, *T.fulvum* differs from *T.parafulvum* by its pileus with radially streaky and relatively larger spores (5.8–7.2 × 4.6–5.1 μm) (Christensen and Heilmann-Clausen 2013). Four species from this section are also found in Shanxi Province, i.e., *Tricholomaaurantium*, *T.ammophilum*, *T.populinum* and *T.viscidum*. Of them, *Tricholomaviscidum* is similar to *T.parafulvum*, both of which are associated with *Quercus* spp. However, *T.viscidum* differs from *T.parafulvum* by its pileus being extremely viscid when wet and relatively larger spores (5–6.5 × 4.5–5.5 μm). The remaining three species are easily distinguished from *T.parafulvum*, *T.ammophilum* and *T.populinum* by their habitats associated with *Populus* spp., *T.aurantium* by its stipe surface covered with dense, orange to brownish orange scaly (Heilmann-Clausen et al. 2017).

#### ﻿ Tricholoma viscidum

Taxon classificationFungiAgaricalesTricholomataceae

L. Fan, N. Mao & J.J Yang sp. nov.

A8B8D722-A97B-54A0-9129-07F1D006BDD6

MB 856308

Figs 2G, H , 3F, H

##### Diagnosis.

It is distinguished by the combination of the following features: pileus surface viscid when wet, stipe surface covered with brown to dark brown fibrils, and broadly ellipsoid basidiospores. It is most similar to *T.ustaloides* Romagn. but differs in the absence of a sharply defined zone of white color on the upper part of the stipe surface.

##### Holotype.

China • Shanxi Province, Qinshui County, Lishan Mountain, 35°29'8"N, 113°1'19"E, elev. 1658 m, on the ground in broadleaf forest dominated by *Quercus* sp., 29 September 2023, J.Z. Cao, MS843 (BJTC FM4198), GenBank Acc. No.: ITS = PQ499539, mtSSU = PQ499571, *tef1-α* = PQ509903, *rpb2* = PQ509913, *mcm7* = PQ509924.

##### Etymology.

*viscidum* (Lat.): referring to the viscid cap of basidiomata when wet.

##### Description.

***Basidiomata*** small, medium to large-sized. ***Pileus*** 33–86 mm diam., at first hemispherical to convex, later plano-convex with age, with an umbo at center; surface viscid when wet, yellowish brown (#e6d3b8), brown (#d0b79d) to reddish brown (#9d6463), darker in the center and paler towards the margin, covered with brown (#ab7d6b) fibrils; margin at first involute, later incurved, cracking with age. ***Lamellae*** sinuate, crowded, dirty white (#f3fbd0) to pale yellow (#f1ffb8), turning brown (#c09a7e) with age; lamellulae in 2–3 tiers, concolorous with lamellae. ***Stipe*** 49–113 mm long, 10–18 mm diam., cylindrical to clavate, enlarged downwards, dry, yellowish brown (#c79f5c) to brown (#9f6327), covered with brown (#b99976) to dark brown (#8b6c68) fibrils. ***Context*** white (#f3eeea), up to 13 mm. ***Odor*** unrecorded. ***Taste*** not recorded.

***Basidiospores*** [120/3/4] 5–6.5 × 4.5–5.5 μm, Q = 1.1–1.25, broadly ellipsoid to ellipsoid, smooth, inamyloid, usually containing one large oil droplet. ***Basidia*** 28–37 × 5–8 μm, clavate, (2–)4-spored, sterigmata up to 5.5 μm long. ***Cystidioid cells*** in hymenium absent. ***Hymenophoral trama*** regular, composed of cylindrical hyphae, 5–10.5 μm wide, colorless in water and 5% KOH. ***Pileipellis*** a cutis, composed of cylindrical hyphae, 4–9.5 µm wide, yellowish-brown in water and 5% KOH. ***Stipitipellis*** a cutis, composed of parallel hyphae, 2.5–6 µm wide, colorless in water and 5% KOH. ***Clamps*** absent in tissues.

##### Ecology and habitat.

Scattered or gregarious on the ground in broadleaf forest dominated by *Quercus* sp., currently only known from Shanxi Province, northern China.

##### Additional specimens examined.

China • Shanxi Province, Qinshui County, Lishan Mountain, on the ground in broadleaf forest dominated by *Quercus* sp., 5 October 2023, J.Z. Cao, CF2268 (BJTC FM4038), GenBank Acc. No.: ITS = PQ499541; ibid., 29 September 2023, J.Z. Cao, MS844 (BJTC FM4199), GenBank Acc. No.: ITS = PQ499542, mtSSU = PQ499572, *tef1-α* = PQ509904, *rpb2* = PQ509914, *mcm7* = PQ509925; ibid., 25 August 2023, H.M. Ji MS443 (BJTC FM3388), GenBank Acc. No.: ITS = PQ499540, mtSSU = PQ499570, *tef1-α* = PQ509902, *rpb2* = PQ509912, *mcm7* = PQ509923.

##### Notes.

*Tricholomaviscidum* belongs to the sect. Genuina (Fig. 1). *Tricholomaustaloides* is closely related to the new species in morphology and phylogeny. They all have ellipsoid basidiospores and are associated with the *Quercus* spp. However, *T.ustaloides* differs from *T.viscidum* by its upper part of the stipe decorated with a distinctly and sharply delimited zone (Christensen and Heilmann-Clausen 2013; Halama et al. 2016). *Tricholomaustale* (Fr.) P. Kumm., a European species often associated with *Fagus* spp. and *Carpinus* spp., is also easily confused with the new species as there are some reports that it is related to *Quercus* forests. However, *T.ustale* differs by its stipe flesh turning reddish brown when cut or bruised and its phylogenetic position (Christensen and Heilmann-Clausen 2013).

## ﻿Discussion

Shanxi Province is located in northern China, where the climate ranges from subtropical to cold temperate. A total of 21 species of *Tricholoma* were confirmed in Shanxi Province in this study (Figs 1–5) based on morphological and molecular data, including four new species described in this paper. They are *T.ammophilum* A.D. Parker, Grubisha & S.A. Trudell, *T.argyraceum* (Bull.) Gillet, *T.atrosquamosum* Sacc., *T.aurantium* (Schaeff.) Ricken, *T.bakamatsutake* Hongo, *T.bonii* Basso & Candusso, *T.boudieri* Barla, *T.cingulatum* (Almfelt ex Fr.) Jacobasch, *T.flavoviride*, *T.frondosae*, *T.fumeobrunneum*, *T.imbricatum* (Fr.) P. Kumm., *T.lishanense* L. Fan & J.J. Yang, *T.parafulvum*, *T.populinum* J.E. Lange, *T.psammopus* (Kalchbr.) Quél., *T.saponaceum* (Fr.) P. Kumm., *T.terreum* (Schaeff.) P. Kumm, *T.triste* (Scop.) Quél., *T.vaccinum* (Schaeff.) P. Kumm., and *T.viscidum*. That means Shanxi Province is rich in species diversity of the genus *Tricholoma*.

Before the present study, there were nine *Tricholoma* species reported in Shanxi Province according to the related professional fungal literatures (Cao et al. 1990; Liu 1991; Mao 2000). Of them, five species are confirmed by the present study, including *T.argyraceum*, *T.cingulatum*, *T.populinum*, *T.terreum*, *T.vaccinum*. Of the remaining four species, *T.gambosum* is a species of *Calocybe* currently as *Calocybegambosa*, which is still not yet confirmed from this province; *T.matsutake* was reported based on the specimens under the tree of *Picea* sp. from Guancenshan Mountain in late 80^th^ of last century (Cao et al. 1990). We are not able to examine the specimens cited by Cao et al. (1990), and also did not obtain new collections, so its occurrence is not confirmed; *T.virgatum* (Fr.) P. Kumm. was reported by Mao (2000), but no specimen was cited. According to the picture of basidiomata cited in his book, we suspect this name is probably misapplied for *T.bonii*; *T.zelleri* (D.E. Stuntz & A.H. Sm.) Ovrebo & Tylutki was reported by Liu (1991) from Qinshui County in southern Shanxi Province under *Pinusarmandii* and *P.tabuliformis*, this name has already been treated as a synonym of *Tricholomafocale* (Fr.) Ricken by Kuo (2018). *Tricholomafocale* is a distinct species, that is morphologically very similar to the new species *T.viscidum* described in the present study, but we did not find it again for the time being.

Many species of *Tricholoma* have been observed having exclusively host association in Shanxi Province in this study; for example, both *T.bonii* and *T.psammopus* are associated with Larixgmelinii var. principis-rupprechtii only, *T.vaccinum* with *Picea* spp. only, *T.populinum* with *Populus* sp. only, *T.terreum* and *T.imbricatum* with *Pinustabuliformis* only, some species, viz. *T.atrosquamosum*, *T.bakamatsutake*, *T.fumeobrunneum*, *T.lishanense* and *T.viscidum* with *Quercus* spp. only. A few species have a relatively wide host range. *Tricholomacingulatum* grows under the trees of *Populus* spp. or *Quercus* sp., *T.aurantium* grows under *Quercus* sp. or *Picea* sp., *T.argyraceum* appears under *Pinustabuliformis*, *P.sylvestris*, *Betula* sp. and *Quercus* sp. These host specificities are a useful guide for identifying the *Tricholoma* in the field.

The distribution of *Tricholoma* is significantly influenced by the geography of their host and the climate in Shanxi Province. There are two main geographic zones to be recognized in Shanxi Province: i) the subalpine zone with vegetation composed of coniferous *Larix-Picea* and broadleaf *Populus-Betula* and with a cold climate, which is mainly located in the mountain area above an altitude of 1600 m and some northern regions; ii) the warm zone with the vegetation composed of conifer’s *Pinus* spp. and broadleaf *Quercus* spp. and with a warm climate, which is mapped in most of the central and southern Shanxi Province. Some *Tricholoma* species are only distributed in the subalpine zone, including *T.bonii*, *T.populinum*, *T.psammopus*, *T.triste* and *T.vaccinum*. Only a few species are observed appearing in both subalpine and warm zones, such as *T.argyraceum*, *T.aurantium*, and *T.cingulatum*. In contrast, the warm zone is rich in species of this genus, with at least 13 species found that are only distributed in this zone, including the most popularly encountered *T.terreum*, which is often confused with the northern *T.bonii*.

The climate also clearly shaped the geographic map of the *Tricholoma* species in this province. One of the good samples is the species *T.bonii*, an exclusive *Larix*-associated species in this province. It is the most popularly encountered mushroom in the forest of Larixgmelinii var. principis-rupprechtii in the northern region, but in the southern area, it is completely absent although there are many plantations of *L.gmelinii* var. principis-rupprechtii colonized for more than 50 years. Another example is *T.lishanense*, one of the most common *Tricholoma* in Lishan Mountain under *Quercus* spp.; its distribution is limited to Zhongtiao Mountains, where there are some subtropical climate areas.

## Supplementary Material

XML Treatment for Tricholoma flavoviride

XML Treatment for Tricholoma fumeobrunneum

XML Treatment for Tricholoma parafulvum

XML Treatment for Tricholoma viscidum

## ﻿Additional information

### Author ORCIDs

Ning Mao https://orcid.org/0000-0003-1564-9446

Jia-Jia Yang https://orcid.org/0000-0003-3668-6107

Yu-Xin Zhang https://orcid.org/0000-0002-6757-1903

Ting Li https://orcid.org/0000-0001-7809-3217

Li Fan https://orcid.org/0000-0001-9887-7086

### Data availability

All of the data that support the findings of this study are available in the main text.
